# Assessing TDApp: An AI-based clinical decision support system for ADHD treatment recommendations

**DOI:** 10.3389/fpsyt.2025.1582746

**Published:** 2025-08-22

**Authors:** Evgenia Baykova, Òscar Raya, Cristina Lombardía, Begoña Gonzalvo, Inés Andreu, David Losada, Tania Falkenhain, Ruth Cunill, Domènec Serrano, David Rigau, David Ramírez-Saco, Beatriz López, Xavier Castells

**Affiliations:** ^1^ Institute of Health Care (ICS-IAS), Girona, Spain; ^2^ Control Engineering and Intelligent Systems (eXiT), University of Girona, Girona, Spain; ^3^ Sant Joan de Deu-Numancia Health Park, Barcelona, Spain; ^4^ Ibero-American Cochrane Center (CCIb), Barcelona, Spain; ^5^ Department of Clinical Pharmacology, Valll d’Hebron Barcelona Hospital Campus, Barcelona, Spain; ^6^ TransLab Research Group, Department of Medical Sciences, University of Girona, Girona, Spain

**Keywords:** Attention defcit hyperactivity disorder (ADHD), recommendation systems, evidence base for decision making, shared decision making, Artificial intelligence (AI), patient empowerment, clinical practice guidelines

## Abstract

**Introduction:**

Clinical practice guidelines (CPGs) have several limitations, namely: obsolescence, lack of personalization, and insufficient patient participation. These factors may contribute to suboptimal treatment recommendation compliance and poorer clinical outcomes. APPRAISE-RS is an adaptation of the GRADE heuristic designed to generate CPG-like treatment recommendations that are automated, updated, personalized, participatory, and explanatory using a symbolic AI approach. TDApp is a clinical decision support system (CDSS) that implements APPRAISE-RS for ADHD.

**Methods:**

Two clinical trials were conducted. In both studies a total of 33 and 32 ADHD patients, respectively, requiring treatment initiation or a major treatment change were enrolled. TDApp recommendations were compared to those of selected CPGs (American Academy of Pediatrics, National Institute for Health and Care Excellence, Spanish Health System, Canadian ADHD Resource Alliance, and the Australasian ADHD Professionals Association) CPGs. The diversity of treatment recommendations was analyzed using Blau’s index. Concordance between TDApp and CPGs recommendations was assessed by calculating the proportion of patients for whom TDApp recommended one drug that was also endorsed by CPGs. Dendrograms were plotted to compare the distance between treatment recommendations as calculated using the NbN nomenclature.

**Results:**

The first study investigated eight methods that differed in how patient and clinician preferred outcomes were handled and the extent to which TDApp tailored the analysis of evidence. The method deemed most suitable was examined in the second study, which found that 50-75% of the patients received at least one favorable treatment recommendation. TDApp evaluated over 10 drugs, including recently marketed ones, with amphetamine derivatives emerging as the most frequently recommended interventions. TDApp generated 8–12 distinct treatment recommendations with a diversity index of 0.70-0.88, which was higher than those of CPGs. The proportion of patients for whom TDApp recommendations overlapped with at least one drug endorsed by CPGs ranged from 21.9% to 100%. Dendrogram analysis revealed that TDApp was positioned on one side of the tree, while CPGs clustered together on the opposite side.

**Conclusions:**

TDApp is an advanced prototype of an CDSS offering automated, participatory, personalized, and explanatory treatment recommendations for ADHD. It represents a promising alternative to CPGs for aiding clinicians and patients in shared treatment decision-making.

## Introduction

As medical research continues to expand, evidence-based medicine (EBM) — a model of care that integrates scientific evidence, clinical expertise, and patient values — has become a cornerstone in bridging the gap between the best available evidence, policy and practice in healthcare (1, 2). Clinical practice guidelines (CPGs) play a pivotal role in EBM, serving as systematic tools for appraising evidence and formulating recommendations. In the context of CPG creation, the Institute of Medicine (IOM) recommends using a series of sequential steps for developing CPGs, including formulating a clinical question using the PICO (Patient, Intervention, Comparison, and Outcome) format, searching for relevant studies, conducting a meta-analysis for each comparison and outcome, determining the confidence (quality) in the evidence of effects, assessing the risk-benefit relationship of each intervention, generating treatment recommendations based on the previous analyses, and assigning strength to these recommendations (3). More specifically, the GRADE (Grading of Recommendations Assessment, Development, and Evaluation) Working Group has developed a structured approach to assessing the certainty of evidence regarding effects and the strength of recommendations, as well as frameworks to support the process of translating evidence into decisions. These frameworks also incorporate the assessment of cost and resource use, the impact on inequity, the acceptability of the intervention, and its feasibility for implementation (4, 5).

Treatment recommendations in CPGs have several limitations. One key issue is obsolescence, with 20% of recommendations becoming outdated within a few years of a CPG’s publication (6, 7). Another limitation is oversimplification, as many guidelines adopt a “one-size-fits-all” approach, overlooking variations in disease severity and patient comorbidities when making recommendations (8–10). A further limitation is lack of prospective validation beyond face validity (11, 12), which is somewhat contradictory because CPGs aim to translate scientific evidence into bedside practice, yet their validity are seldom investigated. Centralization is a concern, as the clinician that makes the decision is not involved in the CPG development and because CPGs are often sponsored by governmental organizations or scientific societies and developed by experts with financial ties to companies that either market products, medical devices or tests related to the disease addressed in the CPGs (13–15). Furthermore, patient involvement in the development of CPGs, while desirable, remains limited (16), and their implementation often lacks formats that facilitate patient participation in shared decision-making (17). This centralization may lead to negative perceptions among clinicians and patients (18), who feel their priorities differ from those of CPG committees. As a result, CPGs may be seen as tools to control costs and reduce clinician autonomy, rather than enhance care (19). Altogether, these shortcomings might explain why studies frequently report clinicians’ non-compliance with CPGs as relatively common (20, 21). This lower compliance with CPGs recommendations has negative clinical consequences, as several studies across different specialties have shown that non-compliance leads to poorer treatment outcomes (22–24). Research has demonstrated that non-adherence to CPGs is linked to higher medical costs resulting from prolonged treatment durations, higher complication rates, and the need for more intensive medical interventions (25).

Our team has developed APPRAISE-RS (Automated, uPdated, Participatory, and peRsonAlISEd treatment Recommender System), which adapts the GRADE heuristic to generate clinical recommendations that are automated, updated, personalized, participatory, and explanatory using a symbolic AI approach (26). According to the patient’s characteristics, APPRAISE-RS identifies the RCTs for which the patient meets the inclusion criteria, applies the findings from these studies on the clinician and patient-preferred outcomes to evaluate the risk-benefit relationship of the investigated interventions, and generates treatment recommendations based on these assessments. Despite the importance of cost in treatment selection, APPRAISE-RS does not currently incorporate cost considerations due to significant price variations both within and across countries (27, 28), nor does it address the intervention’s impact on inequity or its feasibility of implementation, as these factors are more relevant to public health policy and healthcare organization than to clinical practice.

APPRAISE-RS addresses the previously mentioned shortcomings of CPGs. It tackles obsolescence by leveraging an updated dataset of studies and overcomes oversimplification by analyzing evidence from RCTs in which the patient meets the inclusion criteria, thereby generating evidence directly applicable to the individual patient. This is attained by cross-referencing patient characteristics with the inclusion and exclusion criteria of RCTs. Centralization is addressed by making EBM participatory and facilitating shared decision making (29). This is attained through integrating patient and clinician preferences, conducting evidence-based analyses of available treatment options based on these preferences, and generating tailored treatment recommendations at the point of care. Finally, APPRAISE-RS ensures that treatment recommendations are explanatory by producing a therapeutic report that presents the results of the analysis of the evidence for each intervention and critical preference. Other artificial intelligence (AI)-based systems/methods/tools have been explored to address some of the shortcomings of the CPGs, including obsolescence (30), lack of personalization in clinical recommendations (31), and insufficient patient participation (32), however, to our knowledge, no other system besides APPRAISE-RS addresses all of these challenges in such a comprehensive manner.

To test the clinical utility of APPRAISE-RS, we selected attention deficit hyperactivity disorder (ADHD) as a proof of concept before extending the approach to other conditions. APPRAISE-RS/TDApp (TDApp for simplicity, available at https://tdapp.org/en/) is a clinical decision support system (CDSS) that implements APPRAISE-RS for ADHD patients (26). ADHD was selected due to its high prevalence health and its substantial consequences, including academic failure, an increased risk of substance use, accidents, and even mortality (33). Furthermore, evidence suggests that providing suitable ADHD treatment is associated with a reduced risk of accidents (34), academic underachievement (35), and legal issues (36). However, ADHD CPGs are subject to the limitations described above, which may contribute to the observed suboptimal clinician adherence to treatment recommendations (37, 38), significant variability in treatment approaches both within and across regions in numerous countries (39–42), and low patient initiation, adherence, and persistence with prescribed treatments (43, 44).

A series of studies have been designed to validate TDApp. In a preliminary *in silico* validation study with simulated patients (26), TDApp generated more updated and personalized treatment recommendations compared to ADHD CPGs, highlighting its potential to address issues of obsolescence and oversimplification in CPGs. Following this *in silico* study, TDApp was clinically tested in ADHD patients. This article reports the findings from the first two clinical studies, conducted consecutively between 2020 and 2024, which aimed to compare TDApp treatment recommendations with those from selected CPGs in ADHD patients. In Study 1, multiple methods for analyzing the scientific evidence and treatment recommendations were studied, with recommendations hidden from clinicians during data collection. In Study 2, a single method was implemented, and TDApp recommendations were made visible to clinicians during clinical visits.

## Study 1

### Methodology:

#### Study design

The study is a non-randomized, non-controlled clinical trial.

##### Patients

The primary objective of the study was to compare TDApp treatment recommendations with those of CPGs by analyzing the diversity and similarity of the recommendations using Blau’s diversity index and dendrograms (see Statistical analysis section). As neither of these methods involves the formulation of an explicit null or alternative hypothesis, a formal sample size calculation was not applicable. Therefore, no power analysis was conducted, and the sample was set at 55 patients. Enrollment began in August 2020 and was halted in May 2022 after 33 patients were included, due to disruptions in standard healthcare operations caused by the COVID-19 pandemic, which impeded normal enrollment during the scheduled data collection period.

The study included male and female patients aged 6–65 years with a DSM-5 diagnosis of ADHD based on clinical assessment, who required initiation or modification of treatment. Exclusion criteria comprised patients already receiving adequate treatment or those requiring minor medication adjustments (e.g., a change in dose or formulation), as well as patients lacking an electronic device with internet access. Adult patients and parents of minors with ADHD provided signed informed consent, while adolescent patients provided assent. Recruitment occurred at 3 study sites in Girona (Catalonia, Spain).

#### Independent variables:

The independent variable was the treatment recommender system for ADHD patients. TDApp was compared with five relevant ADHD CPGs. We included the most recent versions from four internationally recognized institutions with global impact: the American Academy of Pediatrics (45), National Institute for Health and Care Excellence (46), Canadian ADHD Resource Alliance (47), and the Australasian ADHD Professionals Association (48). Also, the CPG from the Spanish Health System (SHS) (49) was included, as the clinical study was conducted in Spain.

TDApp (26) is an AI-based CDSS that generates automated, updated, personalized, participatory, and explanatory treatment recommendations for ADHD patients. The process starts with gathering patient’s demographic and clinical characteristics. Clinicians’ and patients’ or parents’ “preferences” on treatment goals are collected from a list of 18 treatment preferences. Patients and clinicians rate each preference from 1 (not important) to 9 (very important), with preferences rated 7 or higher classified as “critical”. To enhance patient and parent participation, TDApp includes an education module that provides information on ADHD, treatment options and treatment preferences. This module aims to improve health literacy surrounding ADHD and empower patients and parents in the treatment decision-making process by guiding the selection of critical outcomes that TDApp will analyze to assess the risk-benefit relationship of the interventions analyzed. TDApp uses patient characteristics and both patient and clinician critical preferences to formulate PICO questions. In this framework, “P” represents the patient seeking mental health care, “I” corresponds to the pharmacological interventions examined in RCTs, “C” refers to drug vs placebo comparisons, and “O” to the critical preferences selected by the patient and clinician. Next, TDApp answers the PICO question. By selecting RCTs from a curated dataset that match the patient’s demographic and clinical characteristics. TDApp then generates *ad hoc* evidence for each intervention and “critical preference” by applying meta-analysis techniques. The evidence quality is assessed based on the risk of bias (rated from “high” to “very low”). Using the selected critical efficacy and safety outcomes, TDApp makes the risk-benefit relationship judgment based on predefined rules (26) that can be summarized with the following rule of thumb: an intervention has a suitable risk-benefit relationship if, compared to a placebo, it improves the majority of critical efficacy outcomes and does not increase the risk of any critical safety outcome or treatment discontinuation. Conversely, an intervention has an unsuitable risk-benefit relationship if it fails to improve the majority of critical efficacy outcomes and increases the risk of any critical safety outcomes or treatment discontinuation. In all other cases, the risk-benefit judgment is considered “neither favorable nor unfavorable”. Simultaneously, the quality of this judgment is rated from “high” to “low”. Finally, a clinical recommendation is generated from the risk-benefit judgement and its quality, categorized as “strongly in favor”, “weakly in favor”, “weakly against”, or “strongly against” the intervention.

As this was the first TDApp clinical study, treatment recommendations were withheld from clinicians until data collection was completed. Eight methods were employed to integrate patient and clinician critical preferences and to group RCTs into meta-analysis. Regarding procedures for combining patient and clinician critical preferences, two methods were used: the “comprehensive” method, which considered all critical preferences selected by either patients and clinicians, and the “conjoint” method, which only used the preferences selected by both the patient and the clinician. With regard to procedures for combining data from different RCTs in the meta-analysis, four levels (L1, L2, L3 and L4) of data pooling were implemented (see ESM 1 and 2 for examples). In L1, a stringent patient-RCT match based on demographic and clinical characteristics was required, and meta-analyses were performed for each drug and dose. As the level of analysis advanced, these criteria were relaxed.

#### Outcome measures:

The study outcomes analyzed in this article are the treatment recommendations provided by TDApp to patients and those outlined by selected CPGs. The degree of concordance between TDApp’s recommendations and the treatment recommended by other CPGs was assessed using a measure of pharmacological distance, derived from the NbN nomenclature (50). This nomenclature categorizes drugs based on their pharmacological targets and mode of action within the central nervous system. The pharmacological distances in our study range from 0 to 33, with larger values observed between drugs that differ in their targets and modes of action. For instance, the pharmacological distance between methylphenidate and dexmethylphenidate, bupropion, atomoxetine, guanfacine and no treatment is 1, 7, 15, 19 and 33, respectively (see ESM 3 for more details and the full distance matrix, and https://tdapp.org/en/resources/ for future updates as new drugs are investigated).

Additionally, data on the treatment prescribed, patient/parent satisfaction with the participation in treatment decision-making, the educational materials, TDApp usability, treatment effectiveness, safety, physiological measures, and patient satisfaction with the treatment were collected and will be reported separately.

#### Procedures:

The study comprised four visits. During the first visit, informed consent/assent was requested. If the patient/parents agreed to participate, sociodemographic and clinical data were collected during the second visit. This included information on the type and severity of ADHD, clinical global impression, the history of pharmacological treatment for ADHD, and comorbidities and their treatment. Additionally, patients were encouraged to use TDApp’s educational material to help them make informed decisions about treatment goals. During the subsequent visit, which occurred 3–5 days later, patients/parents reported their preferences regarding therapeutic goals. At the end of the third visit, clinical investigators prescribed the treatment they deemed most appropriate. Data on patient/parent satisfaction with the health decision-making process, the educational materials, and TDApp usability and simplicity were also collected. The final visit was scheduled for week 3, during which ADHD symptom severity, patient/parent satisfaction with the treatment, and assessments of adverse events were collected.

The study was registered at clinicaltrials.gov (NCT04228094) and obtained approval from the Ethics Committee of Research with Medicines in Girona (Catalonia, Spain).

#### Statistical analysis:

A descriptive analysis was conducted on patients’ preferences, evidence-related variables, and treatment recommendations. The McNemar test was used to compare dichotomous variables, while the Wilcoxon signed rank and Friedman’s tests were employed to assess differences between two and more than two groups, respectively, as the continuous variables were non-normally distributed. To assesses the diversity in treatment recommendations, Blau’s index was calculated. This index captures both the number of categories (variety) and the proportional distribution of elements across those categories. Its values range from 0 (indicating no diversity, where all elements belong to a single category) to 1 (maximum diversity, where elements are evenly distributed across all categories). Furthermore, the proportion of patients for which TDApp recommended a drug that was also recommended by CPGs was determined. Dendrograms were plotted to compare the concordance between TDApp and CPG recommendations by examining the pharmacological distance between the recommended drugs. Dendrograms are hierarchical visual representations that showcase similarities (in treatment recommendations) between elements (in this study, recommender systems) through branching structures based on clustering algorithms. In this study, dendrograms group recommender systems according to varying levels of similarity in their treatment recommendations, as determined by the pharmacological intervention distance matrix. Recommender systems that cluster together at lower levels of the tree provide more similar (concordant) recommendations compared to those clustering at higher levels. All analyses were conducted using data from patients whose preferences were available (N=30), except for the patient baseline description, which included all patients (N=33).

### Results

#### Patients

Thirty-three patients were enrolled, 3 of whom were lost at follow up. **Table 1** shows the patients’ baseline characteristics. Most patients were children/adolescents, male, and had an ADHD combined subtype. Comorbidities were common, with one-third of patients having a comorbid neurodevelopmental disorder. Almost half of the patients were receiving psychological support for ADHD.

**Table 1:** Patient baseline characteristics in Study 1 with age is expressed as median and ADHD symptom severity as mean.

Study 1		
Patient Sociodemographic and Clinical Characteristics	%	Mean/Median
Age		13.0
Child/adolescent	75.8	
Male	69.7	
ADHD subtype:		
Inattentive subtype	30.3	
Hyperactive subtype	9.1	
Combined subtype	57.6	
ADHD symptom severity		33.5
Comorbid Neurodevelopmental Disorder	33.3	
Intellectual deficit	9.1	
Autism spectrum disorder	15.2	
Learning disorder	15.2	
Depressive disorder	9.1	
Anxiety disorder	18.2	
Social anxiety	15.2	
GAD	3.0	
Eating Disorder	3.0	
Bulimia	3.0	
Disruptive, impulse-control, and conduct disorders	21.2	
Oppositional defiant disorder	9.1	
Conduct disorder	18.2	
Tobacco SUD	24.2	
SUD other than tobacco	27.3	
Alcohol SUD	15.2	
Cannabis SUD	12.1	
Opioid SUD	12.1	
Sedative SUD	3.0	
Stimulant SUD	18.2	
Personality disorder	3.0	
Antisocial PD	3.0	
Epilepsy	6.1	
Current treatment with:		
Methylphenidate	3.0	
Antidepressants	6.1	
Benzodiazepines	18.2	
Antipsychotics	12.1	
Anti-OH	3.0	
Opiates	9.1	
Mood stabilizers	3.0	
Anticonvulsants	6.1	
Psychotherapy for ADHD	45.5	
History of therapeutic failure to:		
Methylphenidate	9.1	
Alpha-adrenergic agonists	3.0	
History of serious adverse effects to:		
Methylphenidate	6.1	

GAD, generalized anxiety disorder; PD, personality disorder; SUD, substance use disorder.

#### Preferences

The mean number of preferences rated as critical was high among patients (mean = 9.2) and moderate among clinicians (mean = 4.5), resulting in a mean of 11.0 critical preferences in the comprehensive method and 3.5 in the conjoint method. The most common critical preferences were symptom improvement, clinical impressions regarding efficacy, and safety concerns such as seizures and vasovagal syncope. Among clinicians, the most common preferences were improvement of symptoms, quality of life, vasovagal syncope and seizures. Overall, 31 distinct combinations of critical preferences for 33 patients were considered using the comprehensive method, and 25 using the conjoint method.

#### Meta-analysis and quality

The number of interventions analyzed ranged from 9 to 18 and varied across levels of analysis, while the number of meta-analyses and the number of studies included in each meta-analysis varied depending on both the level of analysis and the method used to handle clinician and patient preferences (ESM 4). The overall quality of the evidence generated through meta-analyses was low. In general, only minor differences in quality were observed based on the level of analysis and the method used to incorporate clinician and patient preferences. The number of quality points lost due to bias and heterogeneity differed across levels and no clear pattern emerged. Imprecision was the main factor contributing to loss of quality points, with the greatest impact observed at the lowest levels of analysis.

#### Recommendations and comparison with CPGs

The proportion of patients receiving treatment recommendations varied widely depending on the method used for combining the critical preferences selected by clinicians and patient/parents, as well as on the level of analysis (ESM 5). Fewer patients received recommendations “in favor” when employing the comprehensive method compared to the conjoint method. In contrast, no clear trend emerged for recommendations “against”. Regarding the level of analysis, higher levels of analysis were generally associated with a higher frequency of recommendations “against” when using the comprehensive method, whereas the conjoint method yielded mixed results.

**Figure 1:**
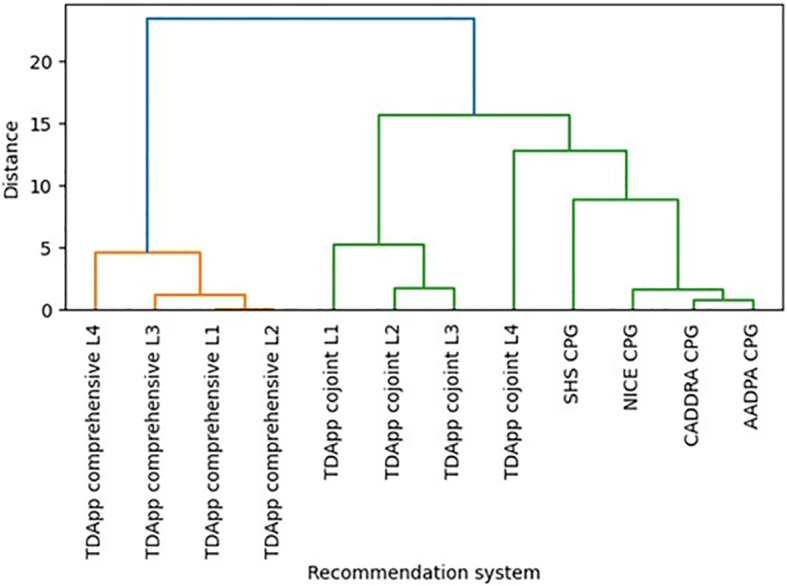
Hierarchical clustering dendrogram depicting similarities on the pharmacological interventions recommended by several versions of TDApp and CPGs in Study 1 (see ESM 12 for an analysis limited to children and adolescents). AADPA, Australasian ADHD Professionals Association; AAP, American Academy of Pediatrics; CADDRA, Canadian ADHD Resource Alliance; CPG, clinical practice guideline; L, level of analysis; NICE, National Institute for Health and Care Excellence; SHS, Spanish Health System.

TDApp considered over 10 drugs, including recently marketed medications such as viloxazine or serdexmethylphenidate, which were not evaluated by any CPG. Methylphenidate and amphetamine derivatives were the most frequently recommended interventions “in favor” (ESM 6 and 7), while atomoxetine and viloxazine were the most frequently recommended “against” (ESM 8 and 9). All treatment recommendations were classified “weak”. The AAP and SHS CPGs recommended both stimulant and non-stimulant drugs, whereas the NICE, CADDRA and AADPA primarily recommended stimulant drugs. The number of distinct recommendations generated with TDApp ranged from 2 to 13, with Blau’s index values ranging from 0.18 to 0.80, which were higher when using the conjoint method (ESM 10). In contrast, CPGs provided 2 to 5 distinct recommendations, with Blau’s index values ranging from 0.17 to 0.50. The proportion of patients for whom TDApp recommended a drug that was also recommended by CPGs was low when using the comprehensive method (ranging from 13.6% to 20.0%) and medium-high (ranging from 56.7% to 95.5%) when using the conjoint method (ESM 11). Dendrograms (**Figure 1**) illustrate that the CPG recommendations, particularly those from NICE, CADDRA and AADPA, clustered together on one side of the tree. In contrast, the recommendations generated by the comprehensive method formed a distinct cluster on the opposite side. Among these, the Level 4 branch connected to the CPG cluster at a shorter distance compared to the Level 1–3 branches. Furthermore, the Level 1–3 branches of the conjoint method clustered together near the center of the figure, while the Level 4 branch was positioned between the remaining conjoint method recommendations and the CPGs — though slightly closer to the latter.

## Study 2

### Methods

#### Study design

The study is a non-randomized, non-controlled clinical trial.

##### Patients

As in study 1, no formal sample size calculation was performed, and the sample was set at 32 patients. The study included male and female patients aged 6 to 17-years with a DSM-5 diagnosis of ADHD confirmed through clinical assessment, who required initiation or modification of treatment. Exclusion criteria included patients already receiving adequate treatment or requiring only minor medication adjustments, as well as those without access to an electronic device capable of connecting to the Internet. Parents provided signed informed consent, while adolescent patients provided assent. Recruitment took place at 2 study sites in Girona (Catalonia, Spain).

#### Independent variables

The independent variable was the recommender system. The recommender systems compared included TDApp, and the CPGs from AAP, NICE, SHS, CADDRA, and the AADPA. In this study, the comprehensive-L3 method was adopted. Although, Study 1 found that the comprehensive method generated treatment recommendations for fewer patients than the conjoint method, the former was chosen for implementation in Study 2 for two reasons: 1) the conjoint method filtered out certain preferences that patients or clinicians deemed critical, which did not fully align with our approach to participatory decision-making. Furthermore, this method could potentially result in no critical preference being used if patients and users did not agree on any of them. 2) The lower number of patients receiving treatment recommendations with the comprehensive method in Study 1 was attributed to the large number of critical preferences TDApp had to consider, resulting in treatment recommendations being identified only for a minority of patients whose treatment goals could be met. To address this surplus of critical preferences in Study 2, patients and clinicians were instructed to be more discriminatory between critical and non-critical preferences, with the recommendation that the number of critical preferences be minimized, with a minimum of one. Level 3 was selected because, as demonstrated in Study 1, when the comprehensive method was used it provided the highest number of distinct recommendations with the greatest level of diversity. Nevertheless, a *post hoc* analysis was conducted to explore the implications of using the conjoint method.

#### Outcome measures

TDApp recommendations were compared to those of the selected CPGs using a measure of pharmacological distance based on the NbN nomenclature (50). Additionally, data on treatment effectiveness on ADHD symptom severity and clinical global impression, adverse events, physiological measures, sense of coherence, self-efficacy, health literacy, patient/parent satisfaction with TDApp, clinicians’ satisfaction with the treatment recommendations, and treatment efficacy and safety were also collected and will be reported in a separate article.

#### Procedures

The study consisted of 5 visits over 6 weeks. During the first study visit, informed consent and assent were requested. If the patient/parents agreed to participate, sociodemographic and clinical history were then collected. Additionally, the patient was encouraged to use TDApp’s educational materials on ADHD and its treatment to make informed decisions about treatment goals. At the next visit, patients/parents and clinicians reported their preferences regarding treatment goals and TDApp generated the treatment recommendations and therapeutic report, which were then presented to the clinician (see ESM 13 for screenshots on TDApp frontend during the study), who prescribed treatment if deemed necessary. During visits 3-5, measures of patient satisfaction were taken of both the educational material and their participation in decision-making, as well as patient empowerment and health literacy, clinician satisfaction with TDApp recommendations, treatment effectiveness and safety, and patient satisfaction with treatment outcomes.

The study was registered at clinicaltrials.gov (NCT05651685) and obtained approval from the Ethics Committee of Research with Medicines in Girona (Catalonia, Spain) before the start of patient enrollment.

#### Statistical analysis:

A descriptive analysis was conducted, and Blau’s index was calculated to assess the diversity in treatment recommendations. The McNemar and the Wilcoxon signed-rank tests were used to compare dichotomous and non-parametric continuous variables, respectively. Dendrograms were generated to evaluate the concordance between TDApp and CPG recommendations by analyzing the pharmacological distance between their suggested treatments.

### Results

#### Patients

Thirty-two patients were enrolled, with one lost to follow up. The cohort was predominantly male, with an average age of 12 years, and exhibited moderate to severe ADHD, predominantly of the combined subtype (**Table 2**). The presence of comorbidities was common, with other neurodevelopmental disorders being the most prevalent. Nearly half of the patients were receiving psychotherapy for ADHD.

**Table 2:** Study 2 patient baseline characteristics.

Study 2		
Patient Sociodemographic and Clinical Characteristics	%	Mean
Age		12.3
Child/adolescent		
Male	65.6%	
ADHD subtype: Inattentive subtype	21.9%	
Hyperactive subtype	0.0%	
Combined subtype	78.1%	
ADHD symptom severity		34.4
Comorbid Neurodevelopmental Disorder		
Intellectual deficit	9.4%	
Autism spectrum disorder	18.8%	
Learning disorder	6.3%	
PTSD	6.3%	
Eating Disorder	3.1%	
Bulimia	3.1%	
Disruptive, impulse-control, and conduct disorders	9.4%	
Conduct disorder	9.4%	
SUD other than tobacco	0.0%	
Current treatment with:		
Antidepressants	6.3%	
Antipsychotics	9.4%	
Psychotherapy for ADHD	46.9%	

GAD, generalized anxiety disorder; PD, personality disorder; PTSD, post-traumatic stress disorder; SUD, substance use disorder.

#### Preferences

The mean number of preferences rated as critical was 4–5 among both patients and clinicians resulting in an average of 6.4 critical preferences analyzed by TDApp per patient. The most common critical preferences were academic performance and improvement of ADHD symptoms on the efficacy side, and seizures and syncope on the safety side among patients, while clinicians prioritized improvement of symptoms, clinical impression, seizures, and appetite decrease. In total, 24 distinct combinations of critical preferences were considered.

#### Meta-analysis and quality:

Around a median of 7–8 interventions were analyzed per patient involving around 29 meta-analyses. The overall quality of the evidence was low, with imprecision being the main reason for reducing the quality of the evidence (ESM 14).

#### Recommendations and comparison with CPGs:

**Figure 2:**
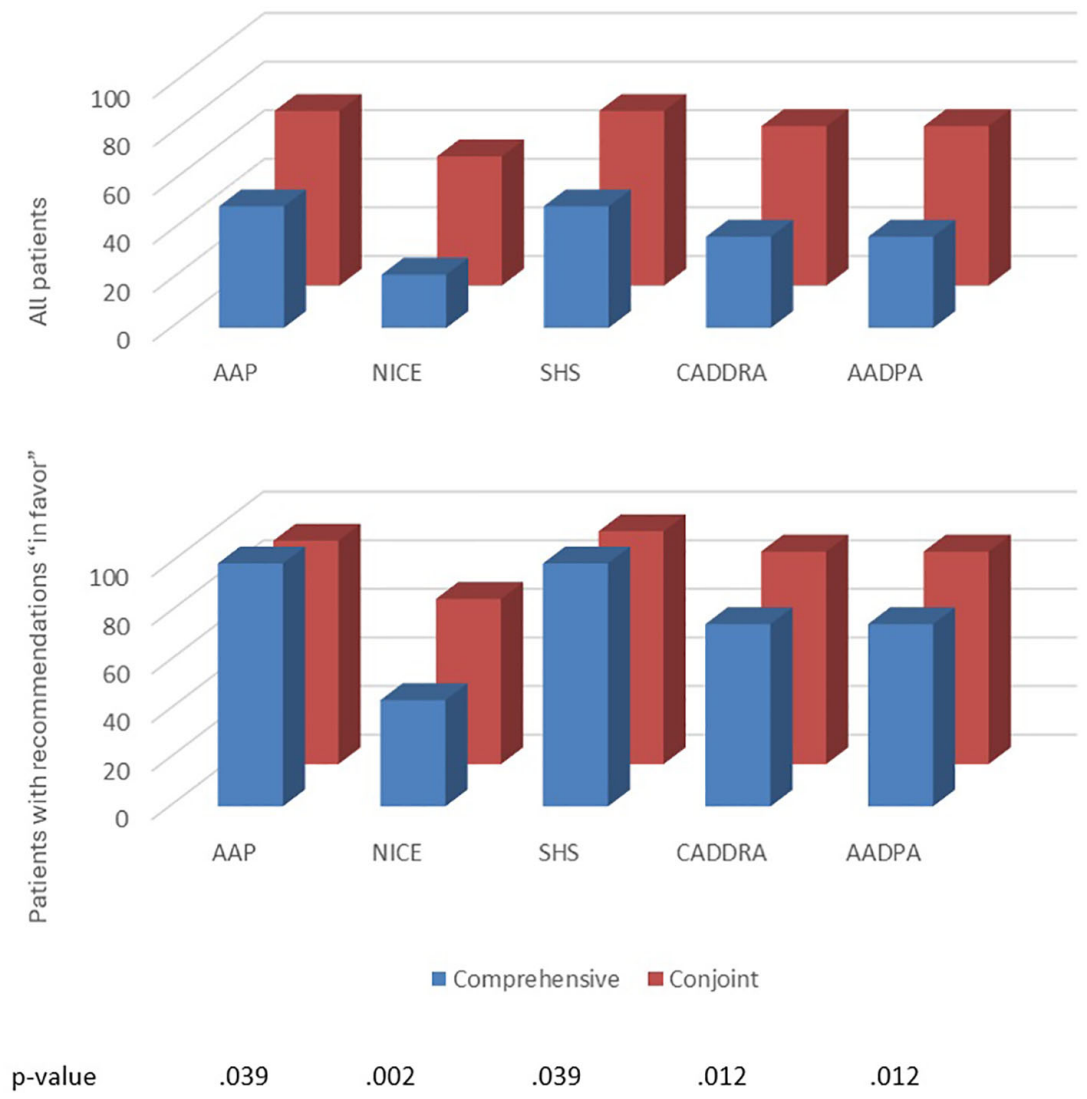
The percentage of patients with at least one overlapping pharmacological treatment recommendation between relevant CPGs and TDApp, using the comprehensive and conjoint methods on all patients (top), and all patients for whom TDApp recommended treatments in favor (bottom) in Study 2. AADPA, Australasian ADHD Professionals Association; AAP, American Academy of Pediatrics; CADDRA, Canadian ADHD Resource Alliance; CPG, clinical practice guideline; L, level of analysis; NICE, National Institute for Health and Care Excellence; SHS, Spanish Health System. The bottom table denotes the p-values for the comparison between comprehensive and conjoint methods for all patients.

**Figure 3:**
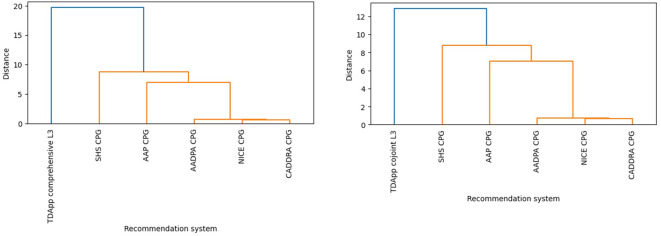
Hierarchical clustering dendrogram depicting differences in the pharmacological interventions recommended by CPGs and TDApp using the comprehensive (left) and conjoint methods (right) in Study 2. AADPA, Australasian ADHD Professionals Association; AAP, American Academy of Pediatrics; CADDRA, Canadian ADHD Resource Alliance; CPG, clinical practice guideline; L, level of analysis; NICE, National Institute for Health and Care Excellence; SHS, Spanish Health System.

Half of patients received at least one treatment recommendation “in favor”, and half received at least one “against” (ESM 15). TDApp generated eight distinct recommendations “in favor” for the study participants, with a Blau’s index of 0.70, while each CPG analyzed generated two distinct recommendations with a Blau’s index of 0.06 (**Table 3**). TDApp considered over 10 drugs, including recently marketed ones. Amphetamine derivatives were the most frequently recommended interventions “in favor”, followed by methylphenidate derivatives and atomoxetine (**Table 3**). Viloxazine, followed by amphetamine derivatives were the interventions most frequently recommended “against” (ESM 16). All treatment recommendations were classified “weak”. The percentage of patients for whom TDApp recommendations overlapped with at least one drug recommended by CPGs ranged from 21.9% with the NICE CPG to 50.0% with the AAP and SHS CPGs. When limiting the analysis to only patients who received a TDApp recommendation “in favor” of treatment, the overlap ranged from 43.8% with the NICE CPG to 100% with the AAP and SHS CPGs (**Figure 2**). Dendrograms (**Figure 3**) showed TDApp recommendations to be positioned on one side of the tree while those from GPGs clustered together on the opposite one with the NICE, CADDRA and AADPA clustering at the lowest levels of the tree. Furthermore, TDApp recommendations from the conjoint method connected at a lower distance to CPGs branches than those form the comprehensive one.

**Table 3:** Comparison of the number, diversity and types of treatment recommended “in favor” by TDApp using the comprehensive and conjoint methods, and relevant CPGs in Study 2.

Number, Diversity and Recommended Treatments		TDApp comprehensive	TDApp conjoint	AAP	NICE	SHS	CADDRA	AADPA
Number of distinct recommendations		8	12	2	2	2	2	2
Blau’s index		0.70	0.88	0.06	0.06	0.06	0.06	0.06
Amphetamine	%	28.1	40.6	0.0	0.0	0.0	0.0	0.0
Atomoxetine	%	15.6	40.6	100.0	3.1	100.0	3.1	3.1
Clonidine	%	0.0	3.1	100.0	0.0	0.0	0.0	0.0
Dexamphetamine	%	28.1	40.6	96.9	0.0	0.0	0.0	96.9
Dexmethylphenidate	%	21.9	46.9	96.9	0.0	0.0	0.0	0.0
Guanfacine	%	6.3	18.8	100.0	3.1	100.0	3.1	3.1
Lisdexamfetamine	%	28.1	40.6	96.9	0.0	96.9	96.9	96.9
Methylphenidate	%	21.9	46.9	96.9	96.9	96.9	96.9	96.9
Mixed amphetamine salts	%	28.1	40.6	96.9	0.0	0.0	96.9	96.9
Serdexmethylphenidate	%	21.9	46.9	0.0	0.0	0.0	0.0	0.0
Viloxazine	%	0.0	6.3	0.0	0.0	0.0	0.0	0.0
Other drugs	%	0.0	15.6	0.0	0.0	0.0	0.0	0.0

AADPA, Australasian ADHD Professionals Association; AAP, American Academy of Pediatrics; CADDRA, Canadian ADHD Resource Alliance; CPG, clinical practice guideline; NICE, National Institute for Health and Care Excellence; SHS, Spanish Health System.

#### *Post hoc* analyses

Upon completion of the primary analysis, a *post hoc* analysis was performed by using the conjoint method for handling patient and clinician preferences. With this approach, the mean number of preferences rated as critical by both the patient and the clinician was 3.2. A median of 7–8 interventions were analyzed per patient involving 22–23 meta-analyses. The overall quality of the evidence was low (ESM 14). Most (75.0%) patients received at least one treatment recommendation “in favor” and 40.6% at least one “against” (ESM 14). TDApp generated 12 distinct recommendations “in favor” (**Table 3**), with a Blau’s diversity index of 0.88. Methylphenidate, amphetamine derivatives and atomoxetine were the most frequently recommended interventions “in favor” (46.9%, 40.6% and 40.6%, respectively), while the interventions most frequently recommended “against” (ESM 16) were viloxazine, atomoxetine and methylphenidate derivatives (25.0%, 12.5% and 6.3%, respectively). Three (9.4%) patients received at least one “strong” treatment recommendation. The proportion of patients for whom TDApp recommendations coincided with at least one drug ranged from 53.1% to 71.9% when considering all samples, and from 68.0% to 95.8% when analyzing only patients with TDApp recommendations (**Figure 2**). The dendrograms (**Figure 3**) reveal that TDApp recommendations are positioned on one side of the tree, while the other systems form a separate cluster on the opposite side.

## Discussion

TDApp is a “first in class” CDSS designed to assist clinicians in shared treatment decision-making by generating up-to-date, personalized, participatory and explanatory treatment recommendations. Findings from the initial clinical study indicate that, while CPGs provided treatment recommendations for 100% of ADHD patients, the proportion of patients receiving recommendations from TDApp varied significantly depending on the methods employed. Modifying the method for matching patients with RCTs and for combining interventions in meta-analysis influenced the number of studies included in the meta-analysis, the evidence generated, its quality, and the number and type of interventions recommended. However, these changes had little impact on the proportion of patients receiving recommendations “in favor”. In contrast, adjustments to the methods for handling critical preferences significantly affected the number of endpoints considered in the evidence analysis. This, in turn, influenced the likelihood of identifying interventions with a favorable risk-benefit relationship, ultimately affecting the proportion of patients receiving recommendations “in favor”. In Study 2, the comprehensive-L3 method was implemented, and patients/clinicians were required to discriminate between critical and non-critical preferences. With this approach, TDApp generated recommendations “in favor” for half of the participants, representing an improvement over Study 1, but still falling short of our expectations. Consequently, the data were re-analyzed using the conjoint method, resulting in “in favor” treatment recommendations for the majority (75%) of patients. Based on these findings, it appears reasonable to implement the comprehensive-L3 method as the default approach while allowing for the use of the conjoint-L3 method when no treatment recommendation can be made. The challenges encountered in developing TDApp highlight its uniqueness as a CDSS designed to generate treatment recommendations, underscoring the complexities of developing participatory decision-making tools. The trial-and-error experience undertaken by the research offers valuable insights for future developers of clinical decision-making tools with a similar approach, potentially facilitating a more efficient path towards an optimal version.

Several findings support the validity of TDApp as a CDSS that makes up-to-date, personalized, participatory and explanatory treatment recommendations for ADHD patients. First, the meta-analyses results and their quality are consistent: higher levels of analyses tend to include more studies, more precise effect estimates, and greater statistical heterogeneity. Second, TDApp found that most meta-analyses were of low quality, which is consistent with other systematic reviews with meta-analysis investigating the efficacy and safety of interventions on ADHD (51–53). Third, the comprehensive method, which incorporates more critical preferences than the conjoint method, tends to generate fewer recommendations “in favor”. Fourth, TDApp fundamentally recommends FDA/EMA ADHD-approved drugs, particularly psychostimulants, aligning with drug prescriptions in clinical practice (54–56). In contrast, atomoxetine was one of the least prescribed interventions, which aligns with evidence indicating that atomoxetine has a questionable risk-benefit profile (51, 52), as well as data on ADHD drug use showing it is prescribed to a minority of patients (42, 54). Furthermore, it is frequently associated with treatment discontinuation and switching after initial prescription (57). Fifth, the proportion of patients for whom TDApp recommendations overlapped in at least one drug with CPGs was notably high. Sixth, since Level 4 provides the most general treatment recommendations, which are more similar to those found in CPGs, its branches in the dendrograms connect to the CPGs branches at a shorter distance than those from the lower levels. Similarly, the recommendations generated by the conjoint method—like CPGs, based on a smaller number of preferences—also cluster closer to the CPGs than those generated by the comprehensive method. Finally, the observation that TDApp does not recommend drug treatment for certain patients further supports its validity, as approximately one in four patients with ADHD are ineligible for RCTs (58).

TDApp offers up-to-date recommendations, demonstrated by its inclusion of newly investigated drugs for ADHD, such as viloxazine and serdexmethylphenidate, which are not included in the latest versions of the selected CPGs. TDApp’s capacity to offer up-to-date treatment recommendations is clinically relevant, as delays in guideline updates is known to be a reason for clinician non-adherence to CPGs (59). Another implication of TDApp up-to-date recommendations is its potential to bridge the gap between cutting edge research and clinical practice, a gap that is particularly pronounced with CPGs due to the time required for their updates.

TDApp makes participatory clinical recommendations by allowing patients to specify the treatment goals they want to achieve. This is particularly relevant as patient involvement has shown to improve treatment adherence in ADHD patients (60). This study provides additional data to gain insight into this issue by revealing large between-patient variability in their preferences, therefore challenging the conventional approach of centralized selection of clinical preferences in CPG development, which fails to account for patient variability in treatment goals. Another important finding related to patient participation is that many patients identified academic performance as a key treatment outcome. This is significant for two reasons. First, none of the selected CPGs considered academic achievement a critical preference, increasing the chances of misalignment between CPG recommendations and patients’ values and preferences. Second, it highlights the need for additional RCTs to assess treatment efficacy on improving academic performance, as this is a demand from patients and parents (61).

Personalization is achieved through an evidence-based analysis of individual patient characteristics and preferences, revealing significant variability and underscoring the need to tailor treatment recommendations to each patient. Unlike CPGs, where PICO questions are formulated by a centralized panel of experts, TDApp, constructs PICO questions based on patient characteristics while also considering critical preferences expressed by both patients and clinicians. Consequently, TDApp generates a broader range of recommendations than CPGs, as shown by a higher number of distinct recommendations and an increased Blau’s index, particularly in Study 2, as well as with the conjoint method in Study 1. Furthermore, the greater diversity of treatment recommendations provided by TDApp can also be attributed to the fact that it considers a wider range of interventions than CPGs. This includes newly marketed drugs, as well as medications approved for indications other than ADHD, such as modafinil or bupropion, though these are infrequently recommended “in favor”.

Finally, similar to CPGs, TDApp provides explanatory treatment recommendations, as each recommendation is supported by an automatically generated evidence-based analysis report, ensuring transparency in the methods used to reach these conclusions. This contrasts with recommender systems, particularly those employing deep learning, which often lack clinical explanations for their recommendations (62). Likewise, systems such as IBM Watson, which uses natural language processing, machine learning, and knowledge-based reasoning, but relies on opaque methods for generating recommendations (63). Both approaches present ethical challenges (64) and limitations in clinical usefulness, as parental trust in treatment decisions increased the likelihood of accepting ADHD medication (65).

It should be noted that the similarity between recommendations provided by TDApp and those from CPGs has been investigated using an *ad hoc* continuous measure of the pharmacological distance based on an external source of information on drug characteristics, namely the NbN nomenclature. This is the first instance of using pharmacological distance, based on an external source, to assess the degree of concordance between different recommender systems. It is noteworthy that that this approach can be implemented in different ways; for instance, our analysis assumes that the difference between two drugs acting on different targets is the same, regardless of the particular targets involved. Thus, a drug acting on serotonin is equally different to two drugs acting on dopamine or on norepinephrine. Future studies would benefit from replicating and modifying our approach in the context of ADHD and other mental health conditions. In addition to this novel approach, a traditional categorical method (66) was also studied by analyzing the proportion of patients who were recommended the same drug. These two methods provide complementary insights. While the quantitative analyses, shown in dendrograms, indicate that TDApp functions as a distinct recommender system when compared to CPGs, the categorical analysis demonstrates that overlap in at least one recommended drug is common, supporting the idea that TDApp is both a distinct and valid recommender system in relation to CPGs.

It is important to highlight that this represents the first step in clinically validating TDApp recommendations. This is a complex issue, as proper validation requires a gold standard for comparison. While ADHD CPGs could be considered this gold standard, CPGs themselves often lack prospective clinical evaluation. Furthermore, different ADHD CPGs may make conflicting recommendations, making it unclear which one should be used as the gold standard for comparison (12). Alternatively, the validity and clinical usefulness of TDApp could be examined through a RCT using clinically relevant outcomes, which should be conducted with the finalized version of TDApp.

The limitations of this study should be emphasized. The sample sizes of both studies are small, though likely adequate to study the concordance between TDApp and CPG recommendations and the differences in their diversity. No formal contrast of hypothesis was conducted to compare the diversity and concordance of recommendations in the dendrograms, as no appropriate methods are currently available. Combinations of drugs were not considered in these studies, but this is of little relevance, as combinations are rarely studied, and no drug combination has received FDA/EMA approval or is recommended by any of the CPGs included in this study. Multiple iterations were explored in Study 1, and *post hoc* analyses carried out in Study 2. This iterative process can result in models overly tailored to the idiosyncrasies of the available data, thereby limiting their generalizability and undermining replicability in broader clinical contexts (64). To prevent this, several transparency practices should be adopted. Protocol registration involves publicly posting a summary of the study’s design, usually including objectives, methodology, and primary outcomes, before the study begins. Study preregistration requires researchers to detail their hypotheses, methods, and analysis plans prior to data collection, enhancing accountability (67). Registered reports go further by having researchers submit a full study protocol for peer review before data collection (68, 69).

TDApp does not take cost into account, despite cost being a significant factor in treatment decision-making. However, this issue can be addressed when implementing TDApp in individual centers and, more specifically, within each country, given that costs vary within and between countries. TDApp recommendations “in favor” were compared to those from CPG; however, we did not compare the recommendations “against” treatment as they are not clearly reported in CPGs. At present, TDApp makes pharmacological treatment recommendations only. Non-pharmacological interventions will be included in the next iteration. Study 1 was conducted during the COVID-19 pandemic, which altered usual healthcare practices and may have introduced potential selection biases. Furthermore, the external validity of the study may be compromised by the fact participants were enrolled in public healthcare centers, which are likely to differ from private healthcare settings.

## Conclusions

- TDApp recommendations vary depending on the methods used for matching patients and RCTs, combining interventions and management of critical preferences.
- TDApp comprehensive-Level 3 and conjoint-Level 3 methods produced diverse and valid treatment recommendations.
- TDApp stands as an advanced prototype of an innovative AI-based CDSS, offering automated, participatory, personalized, and explanatory treatment recommendations. It emerges as a promising alternative to CPGs for assisting clinicians and patients in shared treatment decision-making.

## Data Availability

The raw data supporting the conclusions of this article will be made available by the authors, without undue reservation.
