# Supplementary material for: Assessing TDApp: An AI-based clinical decision support system for ADHD treatment recommendations
Source: Front Psychiatry. 2025 Aug 22;16:1582746. doi: 10.3389/fpsyt.2025.1582746 (PMC12411474; doi:10.3389/fpsyt.2025.1582746)
Supplement: Supplementary file 1 [file SupplementaryFile1.pdf]

Supplementary Materials

ESM 1

Example of how TDApp uses patient characteristics to identify RCTs in which a patient could have participated, based on the 4 levels used. The patient in this example is a 9-year-old girl with a combined subtype ADHD, an ADHD-RS score of 32, and co-morbid dyslexia.

Abbreviations: ADHD = attention deficit hyperactivity disorder, RCT = randomized controlled trial

	**Sex**	**Age**	**ADHD subtype**	**ADHD severity**	**Comorbid diseases**
**LEVEL 1**	Only RCTs accepting girls are selected	Only RCTs accepting 9-year-old patients are selected	Only RCTs accepting patients with a combined ADHD subtype are selected	Only RCTs accepting patients scoring 32 on the ADHD-RS (or equivalent to other scales) are selected	Only RCTs accepting patients with dyslexia are selected
**LEVEL 2**	Only RCTs accepting girls are selected	Only RCTs accepting 9-year-old patients are selected	Only RCTs accepting patients with a combined ADHD subtype are selected	ADHD severity is not an issue	Only RCTs accepting patients with dyslexia are selected
**LEVEL 3**	Only RCTs accepting girls are selected	Only RCTs accepting 9-year-old patients are selected	Only RCTs accepting patients with a combined ADHD subtype are selected	ADHD severity is not an issue	Only RCTs accepting patients with dyslexia are selected
**LEVEL 4**	Sex is not an issue	Only RCTs accepting children or adolescents are selected	ADHD subtype is not an issue	ADHD severity is not an issue	Comorbid disorders are not an issue

ESM 2

Example of how TDApp combines different studies investigating various interventions. This example shows the RCTs that will be combined in a meta-analysis with an RCT investigating methylphenidate 20 mg/d in children with ADHD.

Abbreviations: RCT = randomized controlled trial

**LEVEL 1**	Only RCTs investigating low dose[1] methylphenidate are combined
**LEVEL 2**	Only RCTs investigating methylphenidate are combined
**LEVEL 3**	Only RCTs investigating methylphenidate, dexmethylphenidate and serdexmethylphenidate are combined
**LEVEL 4**	Only RCTs investigating methylphenidate, dexmethylphenidate and serdexmethylphenidate are combined

[1] Low dose methylphenidate: below or equal to 30 mg, 36 mg for OROS methylphenidate, 20 mg for transdermic methylphenidate, 15 mg for dexmethylphenidate, and 45.75mg/9.1 mg for serdexmethylphenidate/dexmethylphenidate (see also López et al., 2023).

ESM 3

This section explains the steps taken to calculate the pharmacological distance between drugs, with the information provided in two tables. The first table lists the pharmacology and mode of action of each drug according to the NbN nomenclature, while the second table shows the pharmacological distance calculated using the information from the first table.

By default, the minimum distance between the two drugs is set at 1. To this base distance, the pharmacological distance is added, which results in two drugs with identical mechanisms of action (methylphenidate and dexmethylphenidate) having a distance of 1. Furthermore, regardless of the drug, the distance between drug and no pharmacological treatment was set at 33.

Pharmacological distance is determined using the NbN nomenclature, which categorizes psychotropic drugs according to their pharmacology (the primary molecular targets or receptors that a drug interacts with to produce its therapeutic effects) and its mode of action (the type of drug interaction with its molecular target leading to clinical effects). According to the NbN nomenclature, the drugs investigated for ADHD act on up to three molecular targets and have up to three modes of action. Bearing this in mind, a weight of 3 is assigned to the first molecular target each drug acts upon, 2 to the second, and 1 to the third. The same approach is applied to the modes of action. The pharmacological distance, defined as the difference in targets and modes of action, was calculated as the sum of the absolute values (Abs) of the differences between each target and mode of action for the two drugs.

For example, methylphenidate targets dopamine and norepinephrine. Its mode of action is multimodal, acting as both a dopamine and norepinephrine reuptake inhibitor and releaser. Accordingly, a 3 is assigned to its target on dopamine and a 2 to norepinephrine, and a 3 to its mode of action as dopamine reuptake inhibitor dopamine and releaser, a 2 to norepinephrine reuptake inhibitor dopamine and releaser, and a 1 for its multimodal modal action. Bupropion targets norepinephrine and dopamine. Its mode of action is norepinephrine and dopamine reuptake inhibitor and release. Therefore, a 3 is assigned to its pharmacology on norepinephrine, a 2 to dopamine, a 3 to its mode of action as norepinephrine reuptake inhibitor dopamine and releaser, a 2 to its role as a dopamine reuptake inhibitor dopamine and releaser and 0 for multimodality. As a result, the pharmacological distance between methylphenidate and bupropion is Abs(3-2)+Abs(2-3)+Abs(3-2)+Abs(3-2)+Abs(2-3)+Abs(2-3)+Abs(1-0)=7.

Note: the average mean between-drug distance is 11.4.

Abbreviations:NA DA = dopamine, inhib = inhibitor, MAO = mono-amine oxidase, NE = norpepinephrine, release = releaser, 5HT = serotonine.

	NE	DA	5HT	NET inhib	DAT inhib	5HT inhib	NE releas	DA releas	NE alpha 2 agonist	DA agonist	MAO-A inhib	MAO-B inhib	5HT agonist	Multimodal
Amphetamine	2	3	0	2	3	0	2	3	0	0	0	0	0	1
Atomoxetine	3	0	0	3	0	0	0	0	0	0	0	0	0	0
Bupropion	3	2	0	3	2	0	3	2	0	0	0	0	0	1
Clomipramine	2	0	3	2	0	3	0	0	0	0	0	0	0	0
Clonidine	3	0	0	0	0	0	0	0	3	0	0	0	0	0
Desipramine	3	0	0	3	0	0	0	0	0	0	0	0	0	0
Dexamphetamine	2	3	0	2	3	0	2	3	0	0	0	0	0	1
Dexmethylphenidate	2	3	0	2	3	0	2	3	0	0	0	0	0	1
Guanfacine	3	0	0	0	0	0	0	0	3	0	0	0	0	0
Lisdexamfetamine	2	3	0	2	3	0	2	3	0	0	0	0	0	1
Methylphenidate	2	3	0	2	3	0	2	3	0	0	0	0	0	1
Mixed amphetamine salts	2	3	0	2	3	0	2	3	0	0	0	0	0	1
Modafinil	0	3	0	0	3	0	0	0	0	0	0	0	0	0
Reboxetine	3	0	0	3	0	0	0	0	0	0	0	0	0	0
Selegiline	2	3	1	0	0	0	0	0	0	0	2	3	0	0
Serdexmethylphenidate	2	3	0	2	3	0	2	3	0	0	0	0	0	1
Viloxazine	3	0	0	3	0	0	0	0	0	0	0	0	0	0

	Amphetamine	Atomoxetine	Bupropion	Clomipramine	Clonidine	Desipramine	Dexamphetamine	Dexmethylphenidate	Guanfacine	Lisdexamfetamine	Methylphenidate	Mixed amphetamine salts	Modafinil	Reboxetine	Selegiline	Serdexmethylphenidate	Viloxazine	no treatment
no treatment	33	33	33	33	33	33	33	33	33	33	33	33	33	33	33	33	33	0
Viloxazine	15	1	11	9	7	1	15	15	7	15	15	15	13	1	14	15	0	
Serdexmethylphenidate	1	15	7	19	19	15	1	1	19	1	1	1	11	15	18	0		

Selegiline	18	14	20	16	14	14	18	18	14	18	18	18	12	14	0
Reboxetine	15	1	11	9	7	1	15	15	7	15	15	15	13	0	
Modafinil	11	13	15	17	13	13	11	11	13	11	11	11	0		
Mixed amphetamine salts	1	15	7	19	19	15	1	1	19	1	1	0			
methylphenidate	1	15	7	19	19	15	1	1	19	1	0				
Lisdexamfetamine	1	15	7	19	19	15	1	1	19	0					
Guanfacine	19	7	17	13	1	7	19	19	0						
dexmethylphenidate	1	15	7	19	19	15	1	0							
Dexamphetamine	1	15	7	19	19	15	0								
Desipramine	15	1	11	9	7	0									
Clonidine	19	7	17	13	0										
Clomipramine	19	9	19	0											
Bupropion	7	11	0												
Atomoxetine	15	0													
Amphetamine	0														

ESM 4

Description of the meta-analyses performed for each patient in Study 1. P-values below "Comprehensive analysis" and "Conjoint analysis" refer to between-level differences, and p-values on the far-right column refer to differences between comprehensive and conjoint methods.

	Comprehensive analysis (Median)	Conjoint analysis (Median)	Median of difference	Percentile 25 of difference	Percentile 75 of difference	p-value
Number of interventions analyzed						
Level 1	18.0	18.0	0.0	0.0	0.0	NA
Level 2	12.5	12.5	0.0	0.0	0.0	NA
Level 3	9.5	9.5	0.0	0.0	0.0	NA
Level 4	17.0	17.0	0.0	0.0	0.0	NA
p-value	<.001	<.001				
Number of meta-analyses performed						
Level 1	77.4	50.5	12.0	2.0	53.0	<.001
Level 2	58.8	43.0	11.0	2.0	44.5	<.001
Level 3	46.0	33.0	8.0	2.0	32.0	<.001
Level 4	95.0	56.0	41.0	17.5	57.5	<.001
p-value	<.001	<.001				
Mean N studies in each meta-analysis						
Level 1	2.9	1.0	1.5	0.1	2.0	<.001
Level 2	3.3	4.2	-0.4	-1.3	0.0	.001
Level 3	5.0	5.4	-0.1	-0.3	0.0	.024
Level 4	10.4	10.7	-0.4	-0.8	0.0	.001

	Comprehensive analysis (Median)	Conjoint analysis (Median)	Median of difference	Percentile 25 of difference	Percentile 75 of difference	p-value
p-value	*<.001*	*.015*				
Mean Overall quality score						
Level 1	2.0	2.0	0.0	-0.5	0.3	.586
Level 2	2.0	2.0	0.1	-0.3	1.0	.011
Level 3	2.1	2.0	0.0	0.0	0.2	.010
Level 4	2.2	2.2	0.1	0.0	0.2	<.001
p-value	*<.001*	*.015*				
Mean bias-related lost points						
Level 1	0.2	0.0	0.1	-0.2	0.2	.957
Level 2	0.0	0.3	-0.3	-0.4	-0.1	<.001
Level 3	0.2	0.2	-0.1	-0.1	0.0	.002
Level 4	0.2	0.3	-0.1	-0.1	0.0	.004
p-value	*<.001*	.092				
Mean heterogeneity-related lost points						
Level 1	0.2	0.0	0.0	-0.1	0.2	.301
Level 2	0.0	0.4	-0.3	-0.5	0.0	.048
Level 3	0.4	0.5	0.0	-0.2	0.0	.006
Level 4	0.4	0.6	-0.2	-0.2	0.0	<.001
p-value	*<.001*	*<.001*				
Mean Imprecision-related lost points						

	Comprehensive analysis (Median)	Conjoint analysis (Median)	Median of difference	Percentile 25 of difference	Percentile 75 of difference	p-value
Level 1	1.7	1.5	0.2	0.0	0.3	.027
Level 2	1.8	1.5	0.0	-0.4	0.4	.926
Level 3	1.4	1.4	0.0	0.0	0.0	.118
Level 4	1.3	1.3	0.0	0.0	0.0	.136
p-value	*<.001*	*<.001*				

ESM 5

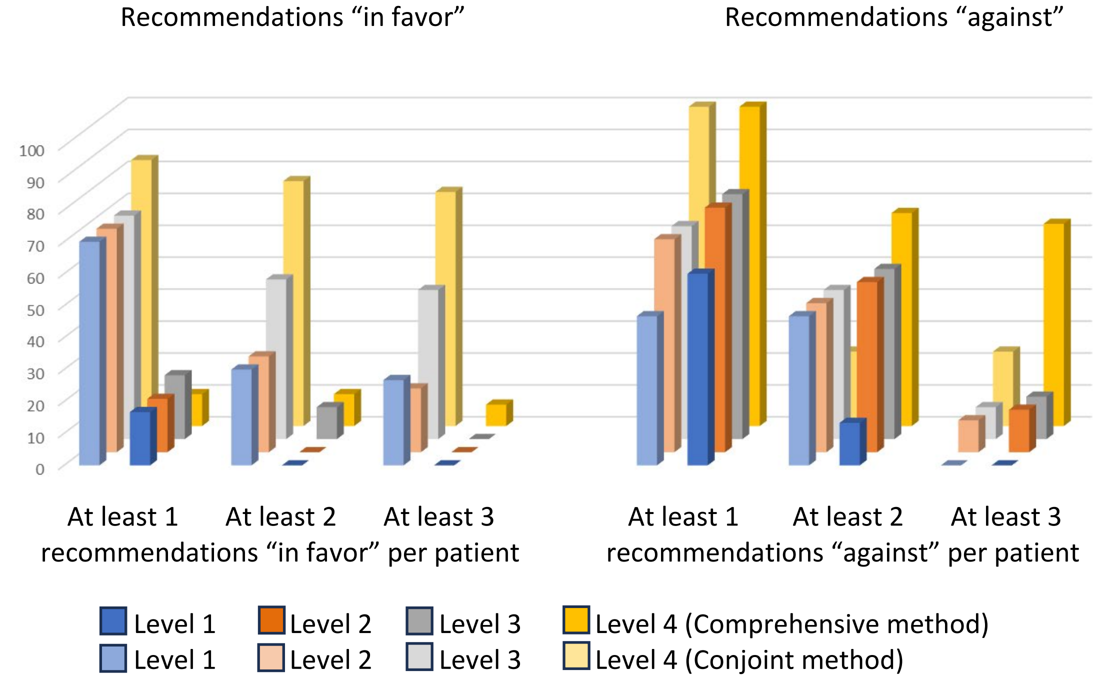

Proportion of patients with at least:

	1 recommendation in favor	2 recommendations in favor	3 recommendations in favor	1 recomendation against	2 recommendations against	3 recommendations against
Level 1	.000	.008	.008	.219	.002	NA
Level 2	.000	.004	.031	.250	.625	1.000
Level 3	.000	.000	.000	.250	.625	1.000
Level 4	.000	.000	.000	NA	.002	.004
Comprehensive	.284	.052	.112	.000	.000	.000
Conjoint	.308	.000	.000	.000	.102	.008

The percentage of patients (%) for whom TDApp made "at least 1", at "least 2" and "at least 3" recommendations "in favor" (on the left) and "against" (on the right) when combining clinician and patient critical preferences using the comprehensive method (bright colors) and using the conjoint method (pale colors) at each level of analysis (blue for "Level 1", brown for "Level 2", grey for "Level 3" and orange for "Level 4").
At the bottom of the panel, the p-values for between-method and between-level comparisons within each level and method, respectively, are displayed. For example, the p-value for the comparison of the proportion of patients with at least 3 recommendations "in favor" between the comprehensive and conjoint methods using Level 1 analysis was .008. In contrast, the p-value for the comparison of the proportion of patients with at least 3 recommendations "in favor" across Levels 1, 2, 3 and 4 using the comprehensive method was .112.
NA = Not applicable

ESM 6

Frequency of recommendations “in favor” by TDApp Level 1 (comprehensive and conjoint methods) and CPGs in Study 1.

	Comprehensive analysis	Conjoint analysis	AAP	NICE	SHS	CADDRA	AADPA
	%	%	%	%	%	%	%
Amphetamine (low dose)	0.0	0.0	0.0	0.0	0.0	0.0	0.0
Amphetamine (high dose)	0.0	0.0	0.0	0.0	0.0	0.0	0.0
Atomoxetine (low dose)	0.0	16.7	100	6.1	100	0	0
Atomoxetine (high dose)	0.0	13.3	100	6.1	100	0	0
Clonidine (low dose)	0.0	0.0	100	0	0	0	0
Clonidine (high dose)	0.0	0.0	100	0	0	0	0
Dexamphetamine (low dose)	0.0	0.0	100	0	0	0	100
Dexamphetamine (high dose)	0.0	0.0	100	0	0	0	100
Dexmethylphenidate (low dose)	0.0	6.7	91.7	0	0	0	0
Dexmethylphenidate (high dose)	0.0	10.0	91.7	0	0	0	0
Guanfacine (low dose)	0.0	10.0	100	3.0	97.0	0	0
Guanfacine (high dose)	0.0	16.7	100	3.0	97.0	0	0
Lisdexamfetamine (low dose)	0.0	10.0	100	24.2	100	100	100
Lisdexamfetamine (high dose)	0.0	10.0	100	24.2	100	100	100
Methylphenidate (low dose)	0.0	20.0	91.7	90.9	90.9	90.9	90.9
Methylphenidate (high dose)	16.7	60.0	91.7	90.9	90.9	90.9	90.9
Mixed amphetamine salts (low dose)	0.0	16.7	100	0	0	100	100
Mixed amphetamine salts (high dose)	0.0	13.3	100	0	0	100	100
Serdexmethylphenidate (low dose)	0.0	0.0	0	0	0	0	0
Serdexmethylphenidate (high dose)	0.0	0.0	0	0	0	0	0
Viloxazine (low dose)	0.0	3.3	0	0	0	0	0
Viloxazine (high dose)	0.0	3.3	0	0	0	0	0
Other drugs	0.0	10.0	0	0	0	0	0

ESM 7

Frequency of recommendations in favor by TDApp Levels 2, 3 and 4 (comprehensive and conjoint methods) and CPGs in Study 1. It should be noted that in Levels 3 and 4 methylphenidate derivatives and amphetamine derivatives are analyzed together as a single group, and are recommended "in favor" or "against" as such.

Abbreviations: AAP = American Academy of Pediatrics, CPG = clinical practice guideline, NICE = National Institute for Health and Care Excellence

	Comprehensive analysis			Conjoint analysis			CPGs				
	Level 2	Level 3	Level 4	Level 2	Level 3	Level 4	AAP	NICE	SHS	CADDRA	AADPA
	%	%	%	%	%	%					
Amphetamine	0.0	13.3	10.0	0.0	43.3	76.7	0.0	0.0	0.0	0.0	0.0
Atomoxetine	0.0	0.0	0.0	6.7	6.7	33.3	100.0	6.1	100.0	0.0	0.0
Clonidine	0.0	0.0	0.0	0.0	0.0	10.0	100.0	0.0	0.0	0.0	0.0
Dexamphetamine	0.0	0.0	10.0	13.3	43.3	76.7	100.0	0.0	0.0	0.0	100.0
Dexmethylphenidate	0.0	16.7	10.0	10.0	60.0	76.7	91.7	0.0	0.0	0.0	0.0
Guanfacine	0.0	0.0	6.7	20.0	20.0	63.3	100.0	3.0	97.0	0.0	0.0
Lisdexamfetamine	0.0	0.0	10.0	10.0	43.3	76.7	100.0	24.2	100.0	100.0	100.0
Methylphenidate	16.7	16.7	10.0	60.0	60.0	76.7	91.7	90.9	90.9	90.9	90.9
Mixed amphetamine salts	0.0	0.0	10.0	20.0	43.3	76.7	100.0	0.0	0.0	100.0	100.0
Serdexmethylphenidate	0.0	16.7	10.0	0.0	0.0	76.7	0.0	0.0	0.0	0.0	0.0
Viloxazine	0.0	0.0	0.0	3.3	3.3	0.0	0.0	0.0	0.0	0.0	0.0
Other drugs	0.0	0.0	0.0	10.0	60.0	23.3	0.0	0.0	0.0	0.0	0.0

ESM 8

Frequency of interventions recommended “against” by TDApp comprehensive-L1 and conjoint-L1 in Study 1.

	Comprehensive analysis	Conjoint analysis
	%	%
Amphetamine (low dose)	0.0	0.0
Amphetamine (high dose)	0.0	0.0
Atomoxetine (low dose)	0.0	3.3
Atomoxetine (high dose)	33.3	33.3
Clonidine (low dose)	0.0	0.0
Clonidine (high dose)	0.0	0.0
Dexamphetamine (low dose)	0.0	0.0
Dexamphetamine (high dose)	0.0	0.0
Dexmethylphenidate (low dose)	0.0	0.0
Dexmethylphenidate (high dose)	0.0	0.0
Guanfacine (low dose)	0.0	0.0
Guanfacine (high dose)	6.7	3.3
Lisdexamfetamine (low dose)	0.0	0.0
Lisdexamfetamine (high dose)	0.0	0.0
Methylphenidate (low dose)	0.0	3.3
Methylphenidate (high dose)	23.3	3.3
Mixed amphetamine salts (low dose)	0.0	0.0
Mixed amphetamine salts (high dose)	0.0	0.0
Serdexmethylphenidate (low dose)	0.0	0.0
Serdexmethylphenidate (high dose)	0.0	0.0
Viloxazine (low dose)	0.0	0.0
Viloxazine (high dose)	0.0	0.0
Other drugs	10.0	16.7

ESM 9

Frequency of interventions recommended “against” by TDApp comprehensive-L2,-3,-4 and conjoint-L2,-3,-4 in Study 1.

	Comprehensive analysis			Conjoint analysis		
	Level 2	Level 3	Level 4	Level 2	Level 3	Level 4
	%	%	%	%	%	%
Amphetamine	0.0	10.0	63.3	0.0	0.0	20.0
Amphetamine	0.0	10.0	63.3	0.0	0.0	20.0
Atomoxetine	0.0	53.3	16.7	53.3	53.3	16.7
Atomoxetine	53.3	53.3	16.7	53.3	53.3	16.7
Clonidine	0.0	0.0	0.0	0.0	0.0	0.0
Clonidine	0.0	0.0	0.0	0.0	0.0	0.0
Dexamphetamine	0.0	10.0	63.3	0.0	0.0	20.0
Dexamphetamine	0.0	10.0	63.3	0.0	0.0	20.0
Dexmethylphenidate	0.0	23.3	63.3	0.0	3.3	0.0
Dexmethylphenidate	0.0	23.3	63.3	0.0	3.3	0.0
Guanfacine	0.0	0.0	0.0	6.7	6.7	13.3
Guanfacine	0.0	0.0	0.0	6.7	6.7	13.3
Lisdexamfetamine	0.0	10.0	63.3	0.0	0.0	20.0
Lisdexamfetamine	0.0	10.0	63.3	0.0	0.0	20.0
Methylphenidate	23.3	23.3	63.3	3.3	3.3	0.0
Methylphenidate	23.3	23.3	63.3	3.3	3.3	0.0
Mixed amphetamine salts	0.0	10.0	63.3	0.0	0.0	20.0
Mixed amphetamine salts	0.0	10.0	63.3	0.0	0.0	20.0
Serdexmethylphenidate	0.0	23.3	63.3	0.0	3.3	0.0
Serdexmethylphenidate	0.0	23.3	63.3	0.0	3.3	0.0
Viloxazine	0.0	40.0	96.7	40.0	40.0	96.7
Viloxazine	0.0	40.0	96.7	40.0	40.0	96.7
Other drugs	16.7	16.7	0.0	20.0	60.0	6.7

ESM 10

Number of distinct recommendations formulated by TDApp and CPGs, and Blau's index measure of diversity in Study 1.

Abbreviations: AADPA = Australasian ADHD Professionals Association, AAP = American Academy of Pediatrics, CADDRA = Canadian ADHD Resource Alliance, CPG = clinical practice guideline, L = level of analysis , NICE = National Institute for Health and Care Excellence, SHS = Spanish Health System

Recommender system	N distinct recommendations	Blau's index
Comprehensive analysis		
Level 1	2	0.28
Level 2	2	0.28
Level 3	4	0.34
Level 4	3	0.19
Conjoint analysis		
Level 1	13	0.79
Level 2	11	0.78
Level 3	10	0.79
Level 4	10	0.80
AAP CPG	3	0.17
NICE CPG	5	0.50
SHS CPG	3	0.18
CADDRA CPG	2	0.18
AADPA CPG	2	0.18

ESM 11

Comprehensive Conjoint

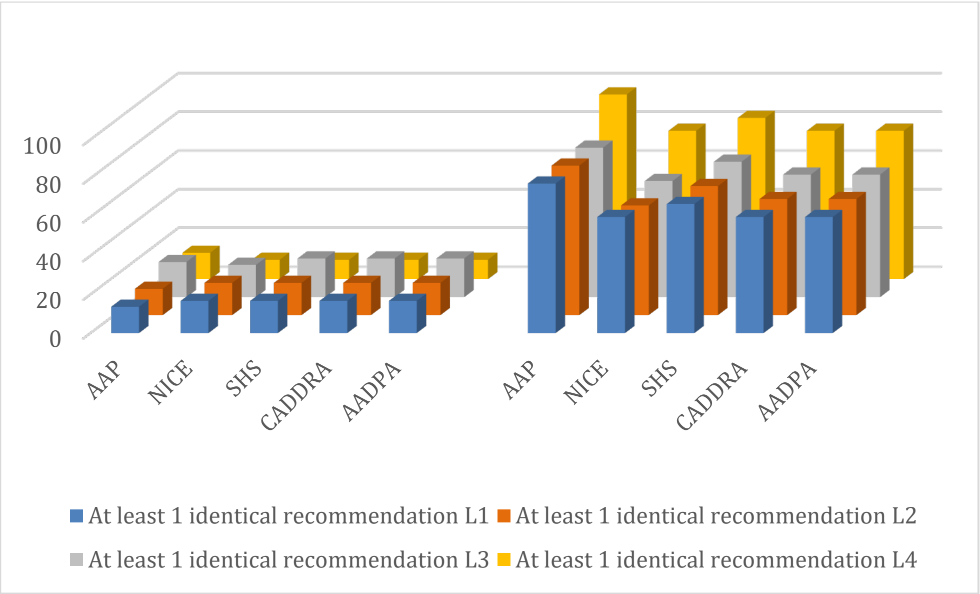

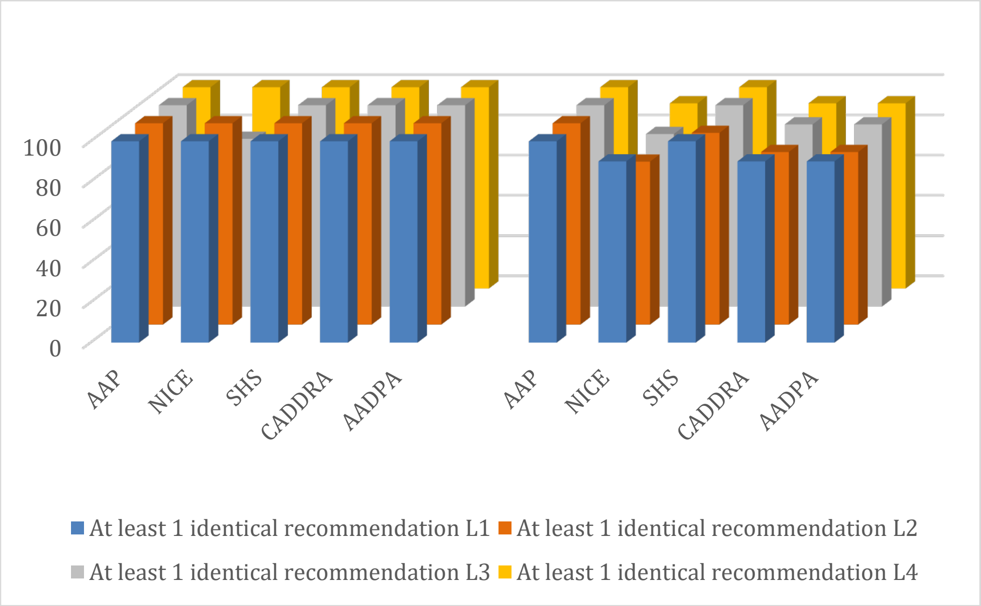

Figure illustrating the percentage of patients with at least one overlapping pharmacological treatment recommendation between TDApp and relevant CPGs, using the comprehensive (left) and conjoint methods (right) on all patients (top), and all patients for whom TDApp recommended treatments in favor (bottom) in Study 1

Abbreviations: AADPA = Australasian ADHD Professionals Association, AAP = American Academy of Pediatrics, CADDRA = Canadian ADHD Resource Alliance, CPG = clinical practice guideline, L = level of analysis, NICE = National Institute for Health and Care Excellence, SHS = Spanish Health System

ESM 12

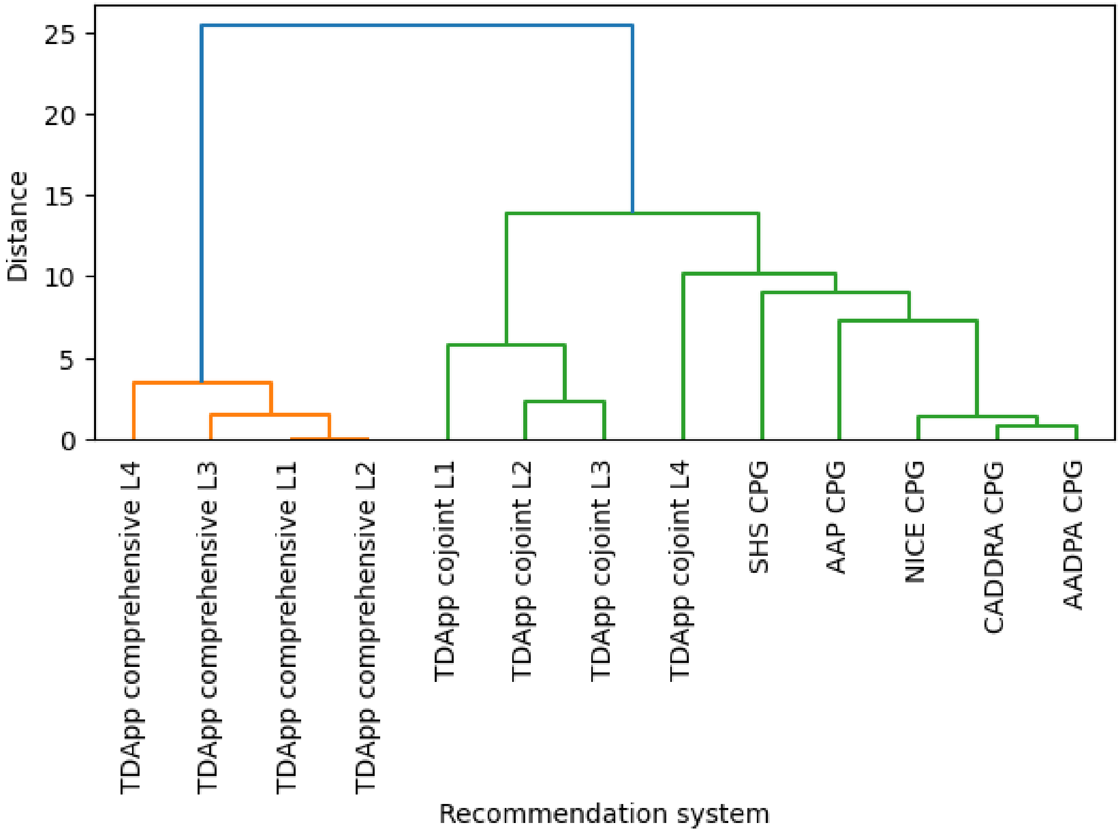

Hierarchical clustering dendrogram illustrating the similarities in pharmacological treatment recommendations made by various versions of TDApp and by CPGs in the subgroup of children and adolescents from Study 1.

Abbreviations: AADPA = Australasian ADHD Professionals Association, AAP = American Academy of Pediatrics, CADDRA = Canadian ADHD Resource Alliance, CPG = clinical practice guideline, L = level of analysis, NICE = National Institute for Health and Care Excellence, SHS = Spanish Health System

ESM 13:

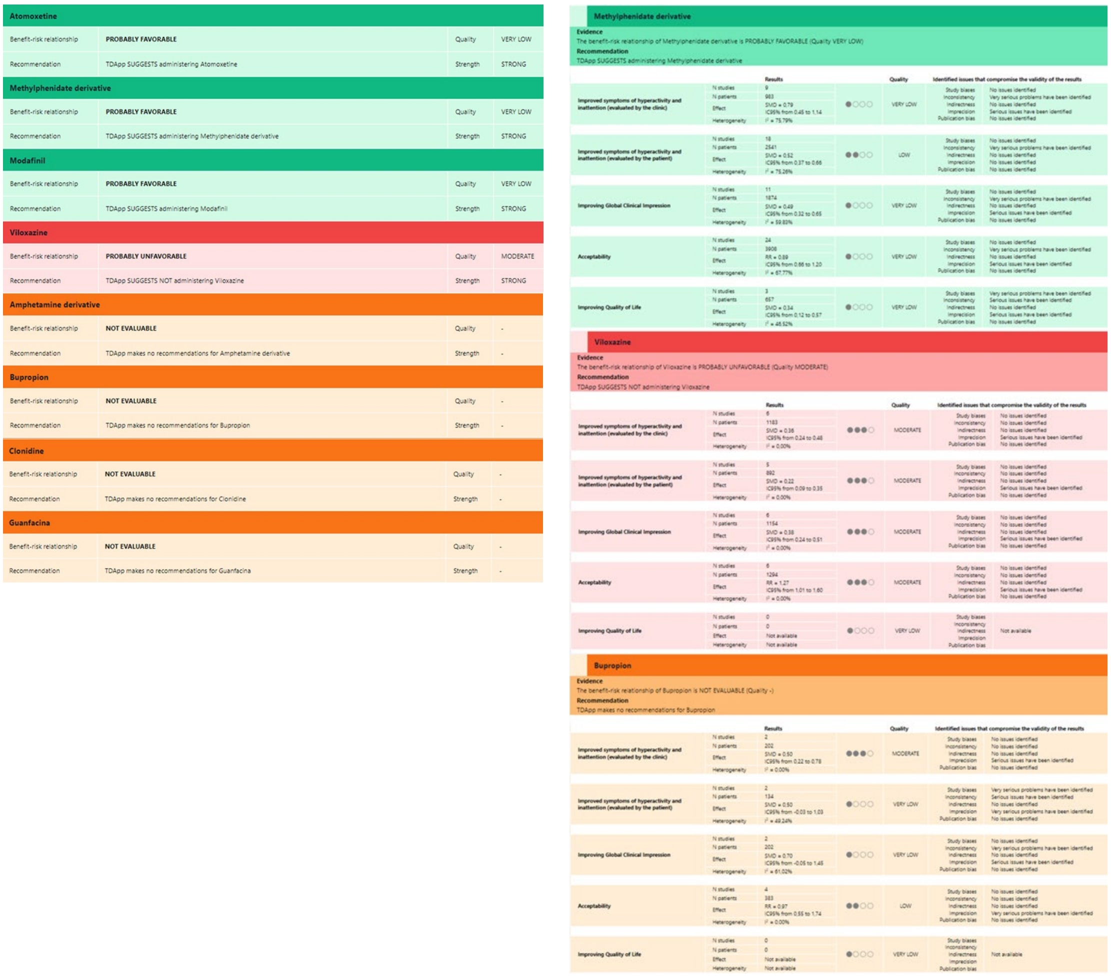

Treatment recommendations generated for a 17-year-old girl with severe ADHD, combined subtype with comorbid depression, where the critical preferences for analysis were ADHD symptom severity improvement, clinical impression, acceptability, and quality of life.

Left panel: treatment recommendations, categorized as “probably favorable” (in green), “probably unfavorable” (in red) or "not evaluable" (in orange).

Right panel: evidence tables that justify these categorizations for each treatment recommendation.

ESM 14

Description of the meta-analyses performed for each patient in Study 2. P-values on the right refer to differences between comprehensive and conjoint methods.

	Comprehensive analysis (Median)	Conjoint analysis (Median)	Median of difference	Percentile 25 of difference	Percentile 75 of difference	p-value
Number of interventions analyzed	7.5	7.5	0.00	0.00	0.00	.157
Number of meta-analyses performed	29.0	22.5	5.50	1.00	12.00	<.001
Mean N analyzed studies in each meta-analysis	5.5	5.6	-0.02	-0.25	0.00	.034
Mean overall quality score	2.2	2.2	-0.07	-0.14	0.00	<.001
Mean bias-related lost points	0.3	0.2	0.00	-0.04	0.05	.520
Mean heterogeneity-related lost points	0.5	0.5	0.00	-0.02	0.07	.224
Mean Imprecision-related lost points	1.1	1.1	0.02	0.00	0.07	.006

ESM 15

Recommendations “in favor” Recommendations “against”

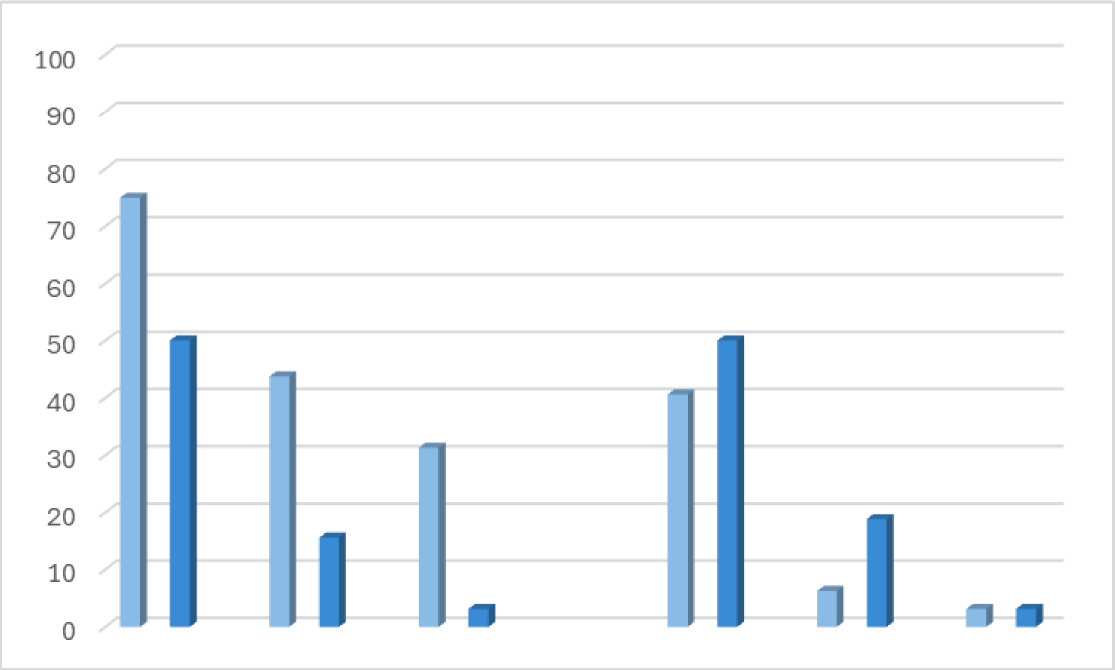

At least 1	At least 2	At least 3	At least 1	At least 2	At least 3
recommendations “in favor” per patient			recommendations “against” per patient		
.021	.004	.004	.453	.219	1.000

Frequency of treatment recommendations in Study 2 using the comprehensive and conjoint methods.

ESM 16

Frequency of pharmacological interventions recommended "against" in Study 2 using the comprehensive (main) and conjoint (*post hoc*) methods.

	Comprehensive	Conjoint
Amphetamine	21.9%	3.1%
Atomoxetine	9.4%	12.5%
Clonidine	0.0%	0.0%
Dexamphetamine	21.9%	3.1%
Dexmethylphenidate	12.5%	6.3%
Guanfacine	3.1%	3.1%
Lisdexamfetamine	21.9%	3.1%
Methylphenidate	12.5%	6.3%
Mixed amphetamine salts	21.9%	3.1%
Serdexmethylphenidate	12.5%	6.3%
Viloxazine	25.0%	25.0%
Other drugs	0.0%	3.1%
